# Dental Erosion in Scrofula

**Published:** 1886-08

**Authors:** 


### Dental Erosion in Scrofula.

M. Eyssantier does not regard dental erosions as due to any specific malady. In this regard he differs from M. Parrot, who taught that syphilis was operative, and from M. Magitot, who considers that infantile convulsions are causative. M. Eyssantier thinks that dental erosions are due to a general innutrition of the organism, which may be brought about by any means, such a scrofula. Scrofula is most active in altering the teeth during the period of second dentition. According to Eyssantier, erosion in furrows of the premolars and molars is most characteristic of scrofula.

